# A Rare Case of Chronic Small Bowel Pseudo-Obstruction

**DOI:** 10.7759/cureus.8003

**Published:** 2020-05-07

**Authors:** Joyce Lim, Daniel Ashmore, Chitakattil Oommen

**Affiliations:** 1 General Surgery, University of Sheffield, Sheffield, GBR; 2 General Surgery, Hull and East Yorkshire Hospitals National Health Service Trust, Hull, GBR; 3 General Surgery, Rotherham National Health Service Foundation Trust, Rotherham, GBR

**Keywords:** chronic intestinal pseudo-obstruction, cipo, pseudo-obstruction, small bowel

## Abstract

Chronic small bowel pseudo-obstruction is rare, and the disease process is poorly understood. Its clinical picture and radiographic findings can resemble mechanical small bowel obstruction and may lead to unnecessary surgery. We report a case of a 68-year-old man who presented acutely with severe abdominal distension and pain after a recent laparoscopic adhesiolysis. His abdominal CT scan revealed grossly distended small bowel with pneumatosis intestinalis and free intraperitoneal air, which led to an exploratory laparotomy. He had a history of having undergone numerous radiological and endoscopic investigations and multiple laparotomies/laparoscopic procedures but without a definitive diagnosis. Subsequent episodes of small bowel pseudo-obstruction occurred, and he developed intestinal failure. His care required the input of multiple healthcare professionals. He was ultimately referred to the National Intestinal Failure Unit for further assessment and management.

## Introduction

Chronic intestinal pseudo-obstruction (CIPO) is a rare disease characterised by the inability of the intestinal tract to propel its contents, resulting in a clinical presentation very similar to intestinal obstruction, but in the absence of any obstructive lesion in the gut [1-3]. It can affect any segment of the gastrointestinal tract, though the small bowel and large bowel are primarily involved [2]. Patients typically present with recurrent abdominal pain, abdominal distension, constipation and vomiting [1,2]. Diagnosis of CIPO is mainly clinical, complemented by a stepwise approach to exclude any lesion causing occlusion of the intestinal lumen [1-3]. The management of CIPO often involves nutritional support and symptomatic control [1-4]. CIPO is an important cause of intestinal failure, which is associated with high morbidity and mortality [1]. The low awareness of the disease among clinicians and the non-specific symptoms associated with the disease often lead to a delayed diagnosis and unnecessary surgery [1,3,5]. We present a case of a 68-year-old man diagnosed with chronic small bowel pseudo-obstruction (and severe gastrointestinal dysmotility) resulting in intestinal failure.

## Case presentation

A 68-year-old man with a history of abdominal pain of unknown cause despite numerous radiological and endoscopic investigations by a gastroenterologist underwent a laparotomy in 2008. This revealed dilatation of the entire small bowel up to 12 cm until two feet from the ileocaecal valve with the collapsed large bowel; no mechanical cause was found to explain the small bowel distension. He subsequently developed an incisional hernia which was repaired laparoscopically with a large intraperitoneal mesh the following year.

**Figure 1:**
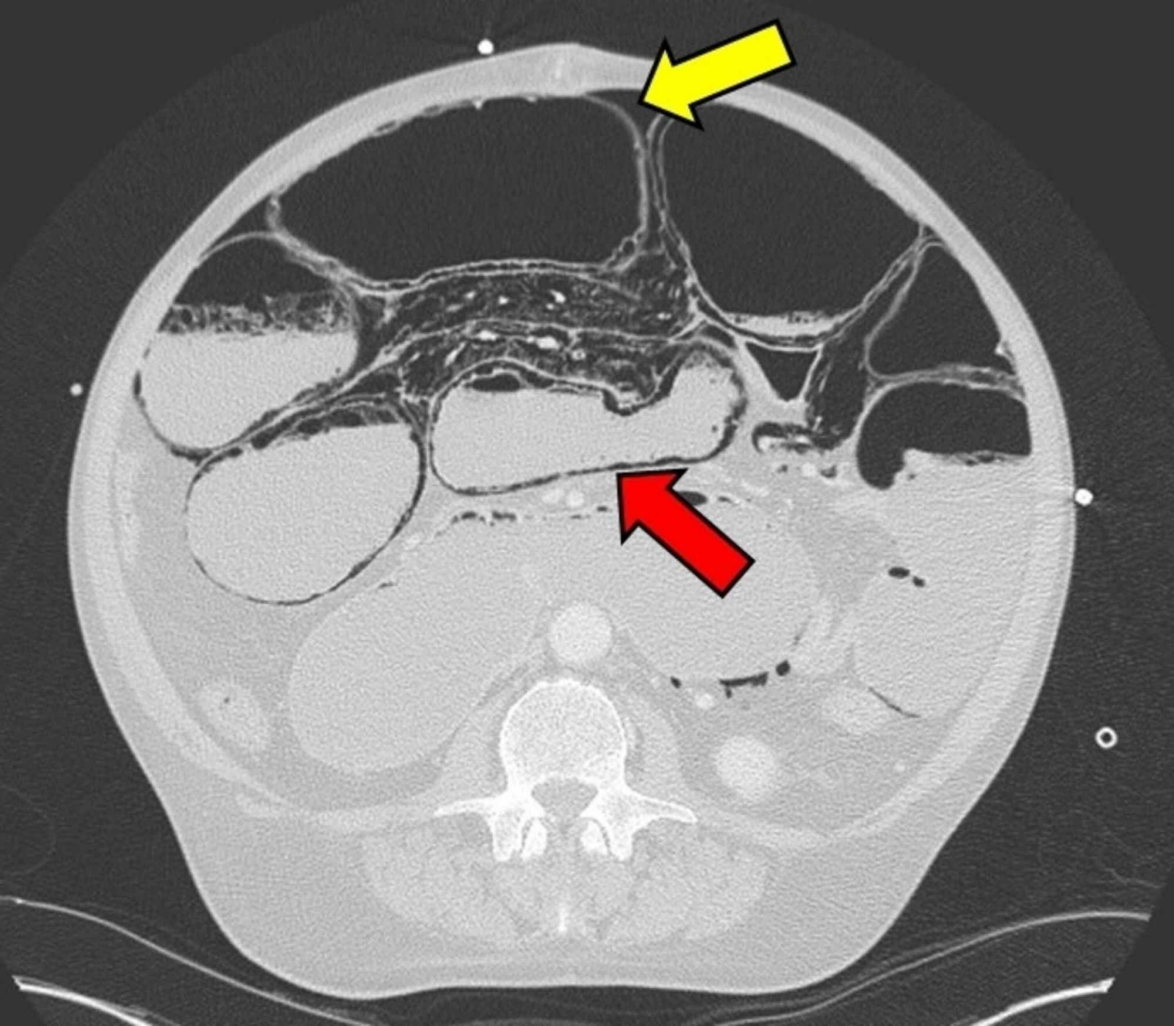
CT scan of the abdomen showing pneumatosis intestinalis (red arrow) and free intraperitoneal air (yellow arrow).

He was readmitted in 2016 and underwent a laparoscopy which showed small bowel distension. Laparoscopic division of adhesions was performed for presumed adhesional small bowel obstruction. After a few days, he developed abdominal pain and distension, and clinical examination showed gross distension of the abdomen with features of peritonitis. CT scan of the abdomen revealed gross distension of the small bowel with pneumatosis intestinalis and free intraperitoneal air (Figure 1). A laparotomy was performed, but it did not show any perforation of the grossly distended (up to 15 cm) entire small bowel; instead gas bubble/sacs were seen in the small bowel wall and the mesentery. Without access to his old notes, the diagnosis was not clear. To decompress the bowel, a double-barrelled ileostomy was fashioned as a venting enterotomy. Over the next few weeks, the abdominal distension reduced, and the stoma started functioning. He was fed enterally. Unfortunately, small bowel stasis and repeated episodes of small bowel pseudo-obstruction resulted in intermittent high output ileostomy and repeated admissions with dehydration and progressive malnutrition. His care proved to be challenging not only to the surgeons but also to the nutrition team. He was referred to the National Intestinal Failure Unit at Salford.

Investigations at Salford Royal Hospital provided further insight. Small bowel manometry showed low amplitude wave for phase III activity in the stomach and duodenum. Barium studies displayed slow propagation of contrast. Large bowel studies were normal, as were investigations for gut hormones, autoantibodies, faecal elastase, amyloid and onconeural antibody screen. The final diagnosis was chronic small bowel pseudo-obstruction. The patient was managed with home parenteral nutrition and after several months, his ileostomy was reversed. He subsequently developed two further episodes of small bowel pseudo-obstruction which were managed conservatively in our hospital over the last two years.

## Discussion

Intestinal pseudo-obstruction refers to a syndrome of disorders which has a similar clinical picture to bowel obstruction, but in the absence of a mechanical obstructive lesion. Its presentation can be acute or chronic [6]. 

The exact prevalence and incidence of CIPO are largely unknown. It is estimated to affect about 100 infants in the United States per year [2]. The estimated prevalence of CIPO in children based on a nationwide study in Japan is 3.7 per 10^6^ children [7]. In adults, the median age of symptom onset is 17 years, with an estimated prevalence of 0.9 per 100,000 people and an average incidence of 0.23 per 100,000 people according to another Japanese survey [8,9]. CIPO is usually idiopathic in children, while in adults, approximately 60% of the cases are secondary to an underlying condition such as scleroderma, systemic lupus erythematosus, dermatopolymyositis, malignancy and amyloidosis [2,3]. Familial forms of CIPO, which may be inherited as autosomal dominant, autosomal recessive or X-linked traits, have also been described [2]. CIPO can also be classified based on the histopathological findings, namely neuropathies, myopathies and mesenchymopathies [1,3]. In adults, neuropathy is more common than myopathy with the latter carrying a poorer prognosis [2,3].

CIPO primarily affects the small bowel and large bowel, though any segment of the gastrointestinal tract can be affected [2]. A study conducted in Chile found that isolated involvement of the small bowel is the most common presentation (59.4%) [10]. Symptoms vary according to the site and extent of the gut segment involved [2]. Symptoms such as nausea, vomiting and weight loss are predominant if the upper gastrointestinal tract is primarily affected, while non-specific abdominal pain, abdominal distension and constipation suggest that a more distal segment of the gut is involved [1]. In adults, onset is generally insidious and may be superimposed with acute episodes, during which symptoms are more pronounced [2]. The severity of symptoms between acute episodes and recurrence rate of these episodes vary from person to person [2].

Several case studies of CIPO involving the small bowel that have been reported in the past are summarised in Table 1. 

**Table 1:** A summary of previous case reports pertaining to chronic intestinal pseudo-obstruction of the small intestine.

Author	Age, sex of patient	Clinical features	Diagnostic tests and results	Treatment and outcome
Naish, Capper and Brown 1960 [11]	36, male	Abdominal pain, abdominal distension, visible and audible peristalsis, steatorrhoea	Laparotomy and biopsy of jejunal wall showed thickened inner muscular coat.	A gluten-free diet was given. Further attacks of pseudo-obstruction occurred and were successfully treated with intravenous fluids and intestinal suction.
Nahai 1969 [12]	19, female	Two-year history of abdominal pain and distension, borborygmi, flatulence and steatorrhoea. The patient also experienced weight loss, ankle swelling and dyspnoea on exertion	Barium meal and radiographs showed grossly dilated small bowel loops up to 10 cm in diameter and fluid levels. Diagnostic laparotomy revealed distended small bowel loops. Full-thickness biopsy of small bowel showed hypertrophy of the inner circular and outer longitudinal muscular coats.	Treatment included antibiotics (ampicillin), drip and suck, parenteral feeding, jejunal and ileal enterostomies. The patient was symptomless postoperatively.
Pelizzo et al. 2013 [13]	14, female	Abdominal distension and severe dehydration	Abdominal radiograph showed small and large bowel dilatation. Exploratory laparoscopy revealed dilatation of ascending colon and terminal ileum. Full-thickness biopsies of the ileum and colon were performed. Immunohistochemistry revealed decreased expression of α-actin in the circular layer of the small bowel.	An ileostomy was performed. A diagnosis of Ehlers-Danlos syndrome (classical type) was made following skin biopsy.
Küllmer et al. 2016 [14]	84, male	Abdominal pain and distension	CT of the abdomen showed massive dilatation of the small intestine and colon.	Treatment included laxatives, prokinetic drugs, endoscopic decompression and percutaneous endoscopic caecostomy (PEC). Postoperative death due to pneumonia was reported.

The diagnosis of CIPO is mainly clinical, supported by dilatation of the bowel and air-fluid level on radiography [1]. The main aims of investigations are to exclude any organic lesion causing occlusion of the intestinal lumen, to identify any underlying illness or reversible cause and to understand the pathophysiological mechanisms which may help to guide management and provide prognostic information [2,3]. A stepwise approach involving radiology, endoscopy, laboratory, manometry and histopathology is recommended [2]. However, the misleading clinical presentation, combined with the limited clinical awareness of this rare disease, often result in a delayed diagnosis and multiple unnecessary surgical interventions [1,3,5]. Moreover, it is not uncommon for patients with CIPO to have concomitant mechanical obstruction due to adhesions secondary to surgery, making it a huge diagnostic challenge [1]. In our case, the patient had previously undergone extensive investigations and surgical procedures for recurrent episodes of abdominal pain, but no definitive diagnosis could be made. His acute presentation of abdominal pain and distension after recent laparoscopic division of adhesions and repeated episodes of small bowel pseudo-obstruction alerted clinicians to a probable diagnosis of CIPO.

Despite recent advances, there is no satisfactory treatment for CIPO. A percutaneous endoscopic gastrojejunostomy or caecostomy may be used as a minimally invasive alternative treatment [14,15]. However, as with our patient, conservatively management is probably the best option unless there is evidence of ischaemia or perforation; this is in line with previous case reports. The long-term treatment for CIPO requires a multidisciplinary approach and often includes pain relief, nutritional support and treatment of complications such as bacterial overgrowth [1-4]. Another important complication of CIPO is intestinal failure, as patients often struggle to maintain their body weight and normal oral nutrition [1,4]. Small bowel involvement, in particular, is associated with more severe nutritional compromise [16]. In the most severe cases, parenteral nutrition may be indicated to ensure adequate nutrition and hydration [2,4].

Our case highlights that CIPO can be a difficult diagnosis; a very high index of suspicion is needed. Early recognition of the disease may help to minimise unnecessary surgical interventions and ensure appropriate management [1]. 

## Conclusions

Pseudo-obstruction involving the small intestine is rare, and the disease process is poorly understood. It should be considered if mechanical causes of intestinal obstruction have been ruled out. Our case illustrates the difficulty in diagnosing and managing CIPO. The intraoperative findings may not concur with preoperative radiological results. The management of this condition is challenging and requires the input of multiple healthcare professionals. A better understanding of the disease process may shed light on more effective treatments of the disease. 
